# Remora Optimization Algorithm with Enhanced Randomness for Large-Scale Measurement Field Deployment Technology

**DOI:** 10.3390/e25030450

**Published:** 2023-03-04

**Authors:** Dongming Yan, Yue Liu, Lijuan Li, Xuezhu Lin, Lili Guo

**Affiliations:** 1 School of Optoelectronic Engineering, Changchun University of Science and Technology, Changchun 130022, China; 2 Zhongshan Institute, Changchun University of Science and Technology, Zhongshan 528400, China

**Keywords:** large-scale measurement field, tooling occlusion, deployment planning, remora optimization algorithm, enhanced randomness

## Abstract

In the large-scale measurement field, deployment planning usually uses the Monte Carlo method for simulation analysis, which has high algorithm complexity. At the same time, traditional station planning is inefficient and unable to calculate overall accessibility due to the occlusion of tooling. To solve this problem, in this study, we first introduced a Poisson-like randomness strategy and an enhanced randomness strategy to improve the remora optimization algorithm (ROA), i.e., the PROA. Simultaneously, its convergence speed and robustness were verified in different dimensions using the CEC benchmark function. The convergence speed of 67.5–74% of the results is better than the ROA, and the robustness results of 66.67–75% are better than those of the ROA. Second, a deployment model was established for the large-scale measurement field to obtain the maximum visible area of the target to be measured. Finally, the PROA was used as the optimizer to solve optimal deployment planning; the performance of the PROA was verified by simulation analysis. In the case of six stations, the maximum visible area of the PROA reaches 83.02%, which is 18.07% higher than that of the ROA. Compared with the traditional method, this model shortens the deployment time and calculates the overall accessibility, which is of practical significance for improving assembly efficiency in large-size measurement field environments.

## 1. Introduction

Research on the deployment planning of digital measuring instruments in large-scale measurement fields mainly focuses on two categories. One is the influence of measurement uncertainty [1] for a specific point on the measurement to determine the interval of the station and optimize the station according to the uncertainty. The other type considers whether the measurement target is measurable as a planning condition and obtains the actual station position of the measuring instrument through accessibility judgment. Accessibility can be divided into two categories: accessibility and visual methods, which are mainly used in the fields of contact and visual measurements. Accessibility analysis [2] is the smallest conical area in a series of observable directions. It is mainly used to analyze whether a target can reach the surface of a measured object without collision and it is suitable for the contact measurement of small and medium parts on a three-coordinate measuring machine. The principle of the visibility graph method [3] is similar to that of the accessibility analysis method. The detection plan derived from the geometry refers to the collection of a series of position points where the optical instrument can measure the light to measure the target point. For the plane measurement point, the visibility graph was a hemisphere collection. A single measuring instrument cannot measure all target points in a large-scale measurement field. Therefore, multiple instruments are needed to work together to build a measurement network covering the entire assembly space. Due to the discrete nature of station locations, Monte Carlo simulation is usually used to solve for the effects of the number of stations, the distance, and the uniformity of distribution on the overall measurement field [4]. However, the reference points to be measured in the traditional method are fixed points, which have certain limitations. Meanwhile, the traditional method of station deployment cannot calculate the overall accessibility. In this study, we transform deployment planning into an optimization problem and obtain the best deployment plan by optimizing the station coordinates using an optimization algorithm with light detection as the rule.

Optimization algorithms can be divided into traditional and metaheuristic optimization algorithms based on the process of solving optimization problems. Meta-heuristic optimization algorithms include evolutionary algorithms, swarm intelligence algorithms, intelligent bionic optimization algorithms, and other intelligent optimization algorithms. In the field of evolutionary algorithms, Dong et al. [5] proposed a novel multi-objective, evolutionary-based, probabilistic transformation inspired by a genetic algorithm. Wan et al. [6] introduced Gaussian chaos mapping and other evolutionary strategies to improve the black widow spider optimization algorithm. Wu et al. [7] combined the Bernstein operator and the differential evolution algorithm and proposed refracted oppositional-mutual learning. Pang et al. [8] used a differential evolution algorithm and multitask learning to predict photovoltaic power. In the field of swarm intelligence algorithms, Opoku et al. [9] combined an ant colony optimization algorithm with iterative conditional patterns for computing estimates of neural source activity. To optimize wireless sensor node deployment, Wu et al. [10] proposed a virtual force-directed particle swarm optimization approach, where the optimization objective is to maximize network coverage. Dai et al. [11] solved the problem of gravity anomaly matching using an artificial bee colony algorithm based on a radiation transformation. Dong et al. [12] combined time-shift multi-scale weighted permutation entropy with a gray-wolf-optimized support vector machine to classify the faults of rolling bearings. In the field of intelligent bionic optimization, Zhou et al. [13] used the immune fruit fly optimization algorithm to search the combined parameters of *k* and *α* in variational mode decomposition. Lu et al. [14] optimized the extreme learning machine for better classification performance using the chaotic bat algorithm. Tong et al. [15] improved the cuckoo algorithm to support continuous hyper-parameters, integer hyper-parameters, and mixed hyper-parameters. Deb et al. [16] commented on the variants and applications of flock optimization algorithms. In other areas of intelligent optimization algorithms, Kuo et al. [17] used simulated annealing to reduce the complexity of a fully connected network. Shang et al. [18] used an artificial immune algorithm to solve the multi-objective clustering problem and to obtain a Pareto optimal solution set. Liao et al. [19] used a firefly algorithm to reduce energy costs. Goh et al. [20] proposed the use of harmony search, to form a hybrid HS-SVM, to perform feature selection and hyperparameter tuning simultaneously and a hybrid HS-RF to tune the hyperparameters.

The remora optimization algorithm (ROA) [21] is a relatively new meta-heuristic optimization algorithm inspired by the parasitic properties of the remora. The algorithm combines the whale optimization algorithm (WOA) [22] and sailfish optimization algorithm (SFO) [23], and the population is updated by switching the two strategies. Almalawi et al. [24] focused on the design of remora optimization and a deep learning heavy metal adsorption rate prediction model for biochar. Raamesh et al. [25] proposed a combination of battle royale optimization and remora optimization to address the selection of software test cases. In this study, different improvements were used. Based on the original ROA, the Poisson-like randomness strategy and enhanced randomness strategy were added such that the population individuals have more changes. In addition, an optimization model was established for the engineering problem of deployment planning in large-scale surveying fields, and high-dimensional parameters were obtained through the improved remora algorithm (PROA) and converted into effective station parameters.

To test the proposed PROA, we used 45 CEC benchmark functions for testing on the base dimension and selected four other meta-heuristic optimization algorithms for performance comparison. Simultaneously, to test the performance of the algorithm in optimizing high-dimensional parameters, we selected 12 CEC benchmark functions with scalable dimensions for testing and comparison. Finally, the improved algorithm was tested and compared to the engineering problem of deployment planning in a large-scale measurement field, and its usability was verified.

## 2. Original ROA

The original ROA was optimized by exploiting the parasitic properties of the remora. Initialization is first performed, and the individuals of the population randomly start their respective initial positions within the upper and lower boundaries. Subsequently, the fitness function of each individual is calculated, and the optimal position and fitness are updated. Attempt a new location using the following formula:

(1) $$R_{att}=R_{i}^{t}+\left(R_{i}^{t}-R_{pre}\right)\times rand_{1},$$

where $R_{att}$ is the attempted new position, $R_{i}^{t}$ is the *i*-th individual in the course of the *t*-th iteration, $R_{pre}$ is the last historical position, and $rand_{1}$ is a normally distributed random number between [0,1]. The fitness $f\left(R_{att}\right)$ of the attempted new position and the fitness $f\left(R_{i}^{t}\right)$ of the current individual are calculated and compared. When the latter is greater than the former, the host feeds as follows:

(2) $$R_{i}^{t+1}=R_{i}^{t}+\left(2V\times rand_{2}-V\right)\times \left(R_{i}^{t}-C\times R_{best}\right)$$

(3) $$V=2\times \left(1-\frac{t}{max\_iter}\right),$$

where $R_{i}^{t+1}$ is the ith individual in the *t*-th iteration process, $R_{best}$ is the global optimal position, $rand_{2}$ is a random number between [0,1], max_iter is the maximum number of iterations, *t* is the current iteration number, *V* is the host feeding range, and *C* is a fixed coefficient of 0.1. Otherwise, the host is changed and the WOA or SFO strategy is used to update the location. The WOA strategy formula is as follows:

(4) $$R_{i}^{t+1}=\left|R_{best}-R_{i}^{t}\right|\times e^{\alpha }\times \cos\left(2\pi \alpha \right)+R_{i}^{t}$$

(5) $$\alpha =rand_{3}\times \left(-\left(1+\frac{t}{max\_iter}\right)-1\right)+1,$$

where $rand_{3}$ is a random number between [0,1] and α is a random number between [−1,1]. The formula for the SFO strategy is as follows:

(6) $$R_{i}^{t+1}=R_{best}-\left(rand_{4}\times \frac{\left(R_{best}+R_{m}^{t}\right)}{2}-R_{m}^{t}\right),$$

where $rand_{4}$ is a random number between [0,1], and $R_{m}^{t}$ is a random individual in the population. Finally, the above steps are repeated until the maximum number of iterations is reached.

## 3. Proposed PROA

### 3.1. Poisson-like Randomness Strategy

**Figure 1:**
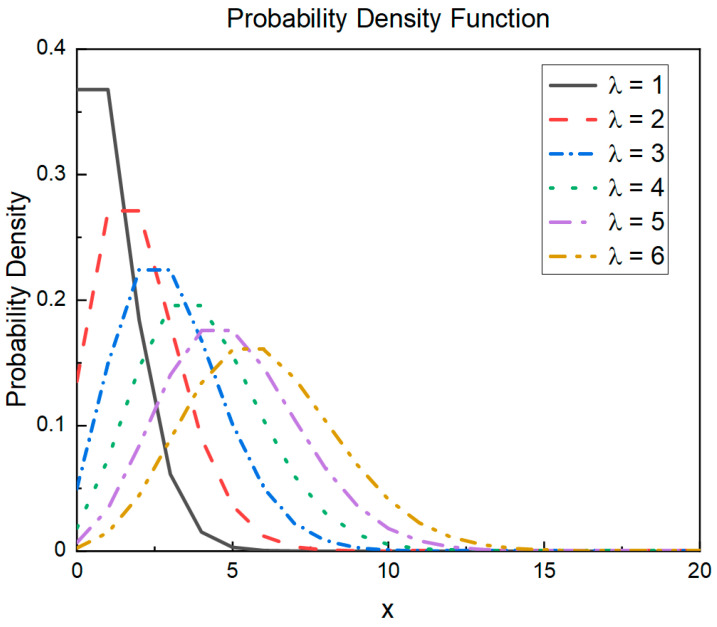
Poisson distribution probability density function.

In the original ROA, a new position was attempted using Equation (1). However, this attempt is only related to the population individuals and their historical positions; the search space is limited, and it easily falls into a local optimum. Therefore, this study introduces a Poisson-like randomness strategy that is obtained by deforming the probability density function of the Poisson distribution. The Poisson probability density function formula is as follows:

(7) $$P\left(X=k\right)=\frac{\lambda^{k}}{k!}e^{-\lambda },$$

where k=0,1,2,…. Figure 1 shows the probability density function curve for λ∈[1,6]. The horizontal axis is *x*, and the vertical axis represents the probability density.

In this study, we set *λ* = 6 for two reasons:

1. The slope was gentle, and there was no sudden change in the function value.
2. The peak and surrounding area are close to one side, which is the opposite of the trend of the change in the strength of the search strategy.

The steps to obtain the two parametric curves of a Poisson-like randomness strategy are as follows:

1. Horizontally mirror the probability density function curve of λ=6 in Figure 1 such that it conforms to the trend of the search strategy strength changes.
2. Parameter curve $r_{1}$ is obtained by stretching the *x*-axis according to the maximum number of iterations of the optimization algorithm.
3. Because the two parameters have opposite trends, $1-r_{1}$ is the parameter curve $r_{2}$.

**Figure 2:**
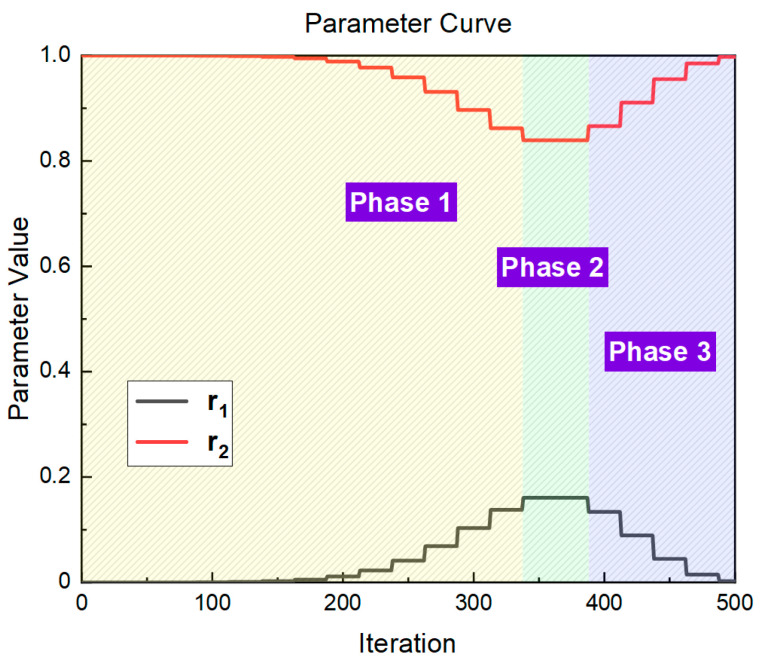
Parameter curve of $r_{1}$ and $r_{2}$.

Considering the maximum number of iterations set to 500 as an example, the two-parameter curves are shown in Figure 2. The entire iterative process is divided into three phases: the yellow area is phase 1, which implies global search; the green area is phase 2, which is close to the optimal solution; and the blue area is phase 3, which implies local search.

Finally, two change parameters were used to adjust the position of other individuals in the population and the optimal position to affect the new position of the attempt. The formula used is as follows:

(8) $$R_{att}=R_{r}^{t}+r_{2}^{t}\times \left(R_{r}^{t}-R_{i}^{t}\right)+r_{1}^{t}\times \left(R_{best}-R_{r}^{t}\right),$$

where $R_{att}$ is the new location attempted and $r_{1}^{t}$ and $r_{2}^{t}$ are the parameter values during the *t*-th iteration.

As shown in Figure 2, the new positions that were tried in the global search phase gradually approached the global optimal solution, and the distance was closest in Phase 2. However, the new locations that were tried during the local search phase were closer to other individuals in the population. From the perspective of the overall search process, Phase 1 enhances the spatial search ability of individual populations. In Phase 3, the individuals are all close to the global optimum, and each individual increases the diversity of local search directions by approaching other individuals.

### 3.2. Enhanced Randomness Strategy

In the original ROA, the SFO strategy was associated with only one individual in the population, and replacement host diversity was not high. Therefore, this study used three enhanced randomness strategies to replace the original SFO strategy. The formula used is as follows:

(9) $$R_{i}^{t+1}=R_{i}^{t}+rand_{5}\times \left(R_{i}^{t}-\left(R_{k}^{t}+R_{h}^{t}\right)/2\right)$$

(10) $$R_{i}^{t+1}=R_{best}^{\;}+R_{d}^{t}+rand_{6}\times \left(R_{e}^{t}-R_{f}^{t}\right)$$

(11) $$R_{i}^{t+1}=rand_{7}\times R_{i}^{t}+rand_{8}\times \left(R_{best}^{\;}-R_{i}^{t}\right),$$

where $R_{k}^{t}$, $R_{h}^{t}$, $R_{d}^{t}$, $R_{e}^{t}$, and $R_{f}^{t}$ are other random individuals in the iterative process and $rand_{6}$, $rand_{7}$, and $rand_{8}$ are random numbers between [0,1].

Compared with the original single strategy, the enhanced randomness strategy strengthens the connection with other individuals in the population, strengthens the connection with the optimal individual, and increases the diversity of the replacement hosts.

### 3.3. Steps to the PROA

**Figure 3:**
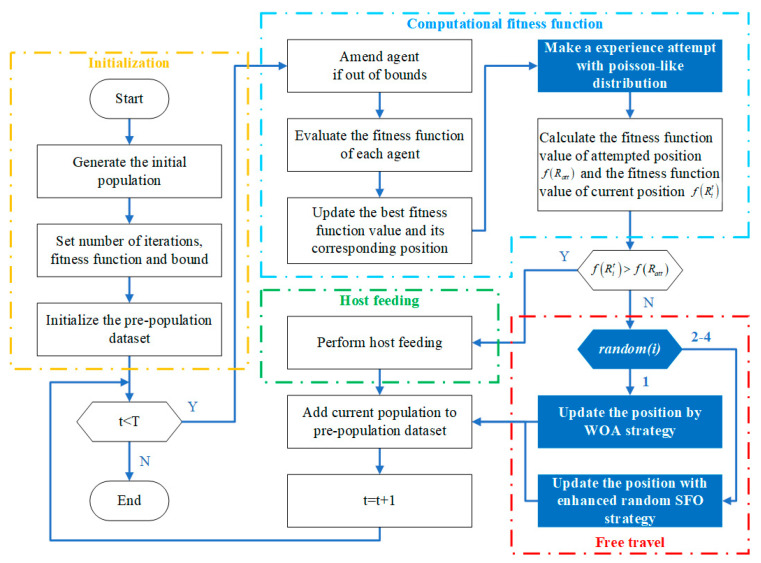
The flowchart of PROA.

In the proposed PROA, the original trial strategy was replaced with a Poisson-like randomness strategy. Simultaneously, the direction of free travel was extended using an augmented randomness strategy. A flowchart is shown in Figure 3, and the pseudocode is presented in Algorithm 1.
**Algorithm 1:** Pseudocode for the PROA.**Input:** population position $R_{i}\left(1,2,\ldots ,n\right)$, the number of iterations max_iter, fitness function f, and bound [lb,ub]. **Output:** best position, best fitness, and fitness history.  1:Initialize the pre-population dataset $R_{pre}$;  2:**While** t<max_iter**carry out**  3: Amend agent if out of bound [lb,ub];  4: Calculate $f\left(R_{i}^{t}\right)$ of each agent;  5: Update $R_{best}$ and $f\left(R_{best}^{t}\right)$;  6: **For** each agent indexed by i **carry out**  7:  Using Equation (8) to make an experienced attempt $R_{att}$ with Poisson-like distribution;  8:  Calculate $f\left(R_{att}\right)$ and $f\left(R_{i}^{t}\right)$;  9:  **If** $f\left(R_{i}^{t}\right)>f\left(R_{att}\right)$
**then**10:   Perform host feeding by Equation (2);11:  **Else**
12:   **If** random(i)=1
**then**13:    Using Equation (4) to update the position by WOA policy;14:   **If**
random(i) in [2,4]
**then**15:    Using Equations (9)–(11) to update the position with enhanced randomness SFO policy;16:   **End if**
17:  **End if**
18:  Add current population to $R_{pre}$;19: **End for**
20: t=t+1;21:**End while**

## 4. Performance Comparison under the CEC Benchmark Function

### 4.1. Experimental Configuration

To examine the convergence speed and robustness of the PROA, we selected 45 benchmark functions proposed in the IEEE CEC competition as fitness functions for testing [26] (refer to the Supplementary Materials for details). In addition, we compared the PROA with the artificial electric field algorithm (AEFA) [27], white shark optimization algorithm (WSO) [28], sooty tern optimization algorithm (STOA) [29], squirrel optimization algorithm (SSA) [30], and the original ROA. In order to ensure the integrity of the six algorithms’ data and to ensure minimum expense, the number of populations is set to 20 and the maximum number of iterations is set to 500. Each algorithm was run 50 times for this configuration. In addition, we selected benchmark functions with 12 scalable dimensions for high-dimensional (D = 100/500/1000) testing with the same configuration as the standard dimensions.

### 4.2. Comparison of Experimental Results

Because of the plethora of CEC benchmark functions for comparison, we present the full experimental results in the Supplementary Materials. In Section 4.2.1, Section 4.2.2, Section 4.2.3, Section 4.2.4, we only present the optimization results of six of the CEC benchmark functions. Information on Rosenbrock (F16) [31], Dixon–Price (F17) [32], Rastrigin (F22) [33], Griewank (F41) [34], Penalized (F43) [35], and Penalized2 (F44) [36] is shown in Table 1.

#### 4.2.1. Comparison of Standard Dimension Results

The experimental results for the standard dimensions are listed in Table 2. From the experimental results, it can be observed that the PROA achieves the best results when optimizing F16, F17, F22, F41, F43, and F44, both in terms of the average result of optimization and robustness. Compared with the ROA, the average value of the PROA increased by two orders of magnitude in the experimental results of optimizing F16, F43, and F44, and the average value also increased by one order of magnitude in the experimental results of optimizing F44. In terms of robustness, the standard deviation of the PROA was reduced to 1% of the ROA in the experimental results of optimizing F16, F17, and F44. In the experimental results of optimizing F43, it was also reduced to 1/20 of the ROA.

**Figure 4:**
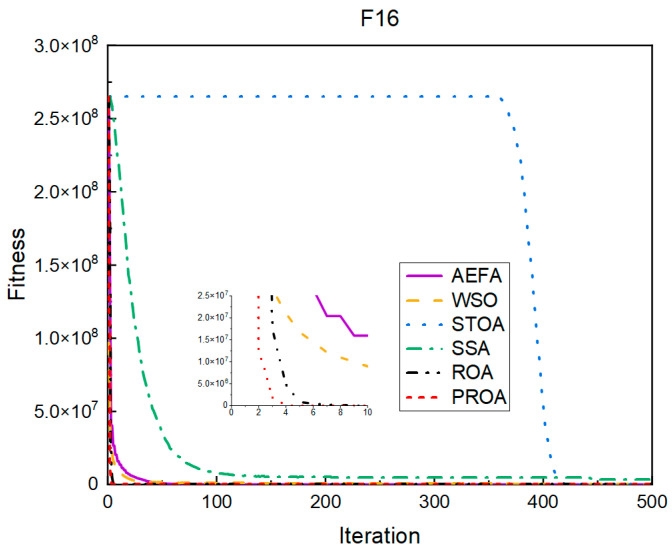
F16’s convergence curve (standard D).

**Figure 5:**
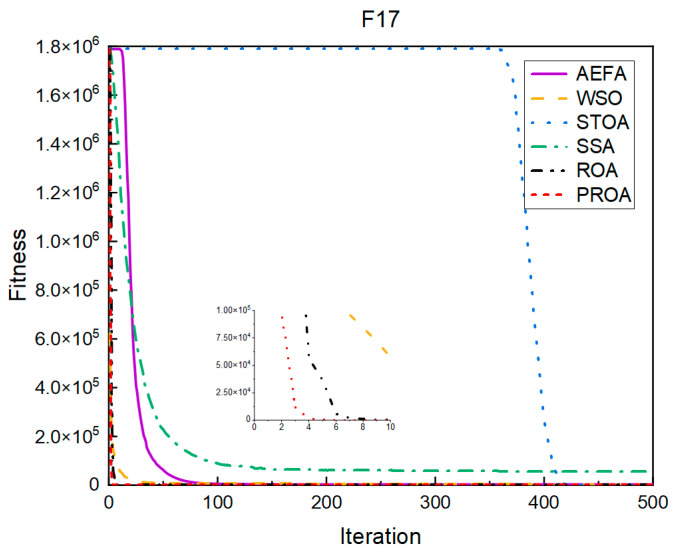
F17’s convergence curve (standard D).

**Figure 6:**
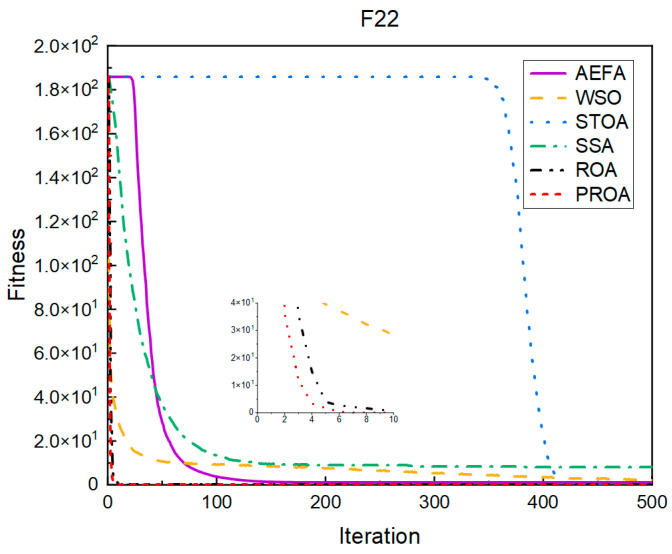
F22’s convergence curve (standard D).

**Figure 7:**
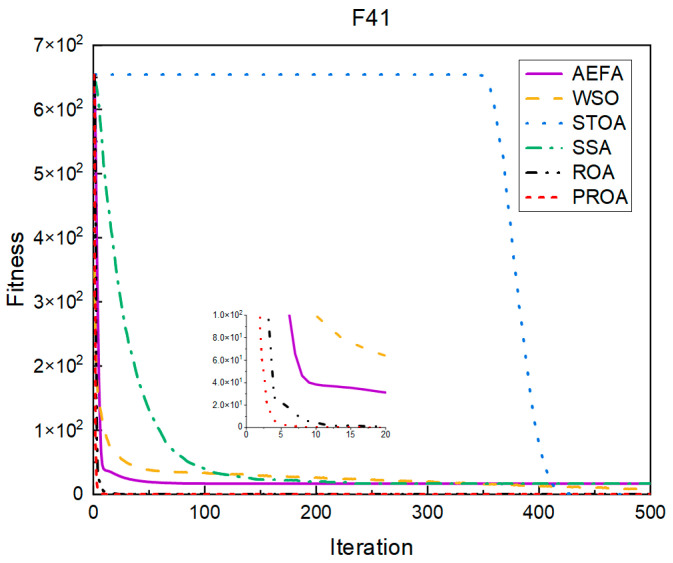
F41’s convergence curve (standard D).

**Figure 8:**
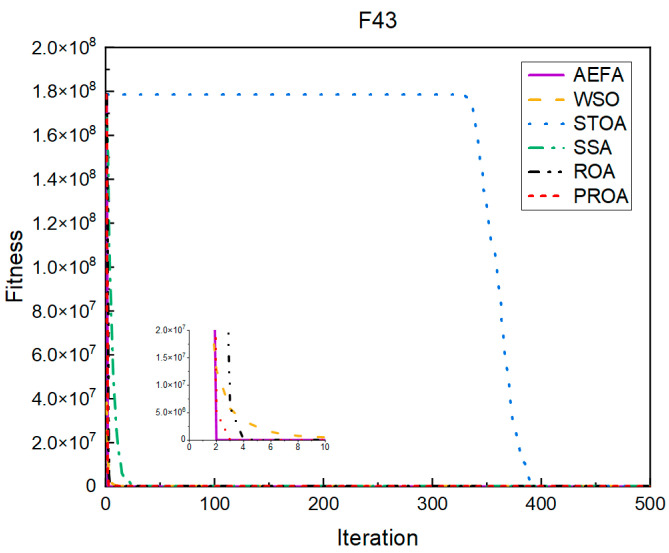
F43’s convergence curve (standard D).

**Figure 9:**
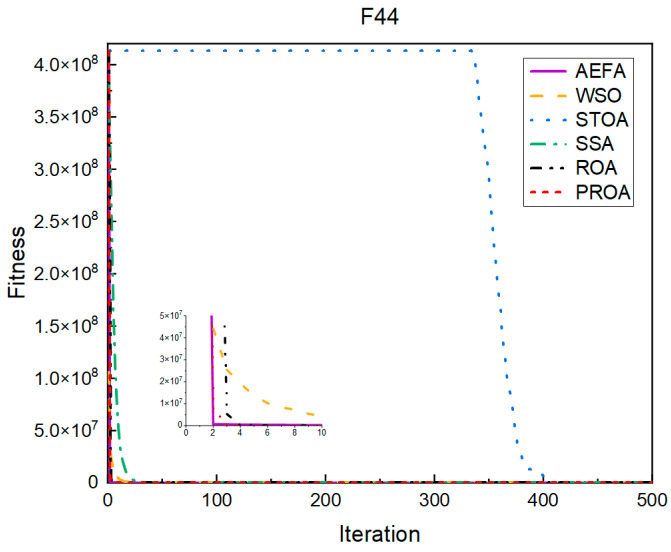
F44’s convergence curve (standard D).

In terms of the convergence speed, the experimental results are shown in Figure 4, Figure 5, Figure 6, Figure 7, Figure 8 and Figure 9. The horizontal axis represents the number of iterations, and the vertical axis represents the fitness function value. It can be observed from the experimental results that, compared with the ROA, the PROA achieves the optimal result twice in advance when optimizing F16, and four times ahead, to obtain the minimum value when optimizing F17, F22, and F41. In the optimization of F43 and F44, the iterative process was also advanced by one round.

#### 4.2.2. Comparison of Results under Dimension 100

The experimental results listed in Table 3 are from when the optimization parameter dimension was 100. From the experimental results, it can be observed that the PROA still performs well in high dimensions. When optimizing F16, the average result of multiple optimizations of the PROA is 0.1% of the ROA, and the standard deviation is 1% of the ROA. However, when optimizing F17, the average result of multiple optimizations is only 1/20 of the ROA. However, its robustness is still 100 times that of the ROA. When the PROA is optimized with F43 as the objective function, the result is 100 times better than that of the ROA regardless of whether the mean or the standard deviation of the optimized results are optimized. When optimizing F44, the result reduces to 0.01% of the ROA result.

**Figure 10:**
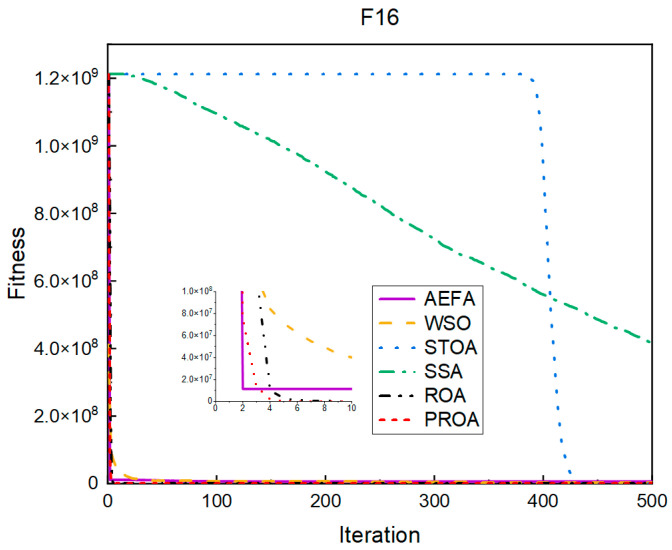
F16’s convergence curve (D = 100).

**Figure 11:**
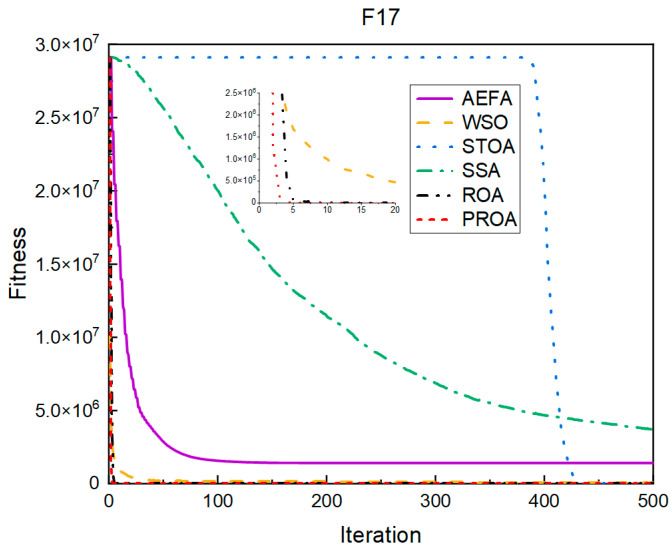
F17’s convergence curve (D = 100).

**Figure 12:**
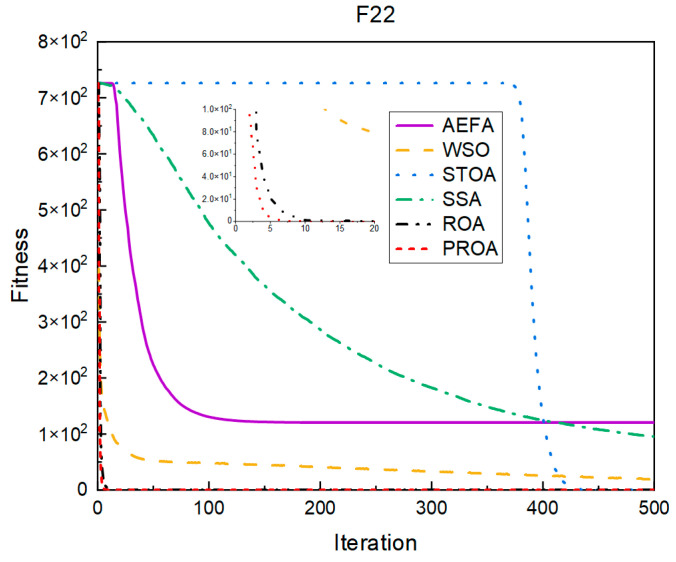
F22’s convergence curve (D = 100).

**Figure 13:**
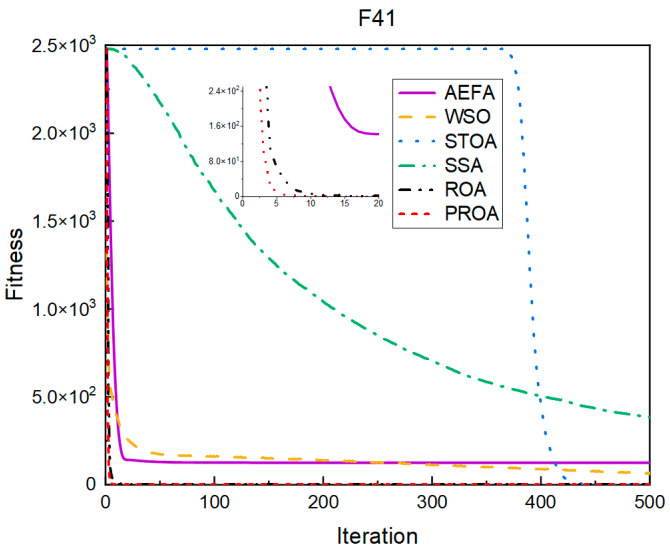
F41’s convergence curve (D = 100).

**Figure 14:**
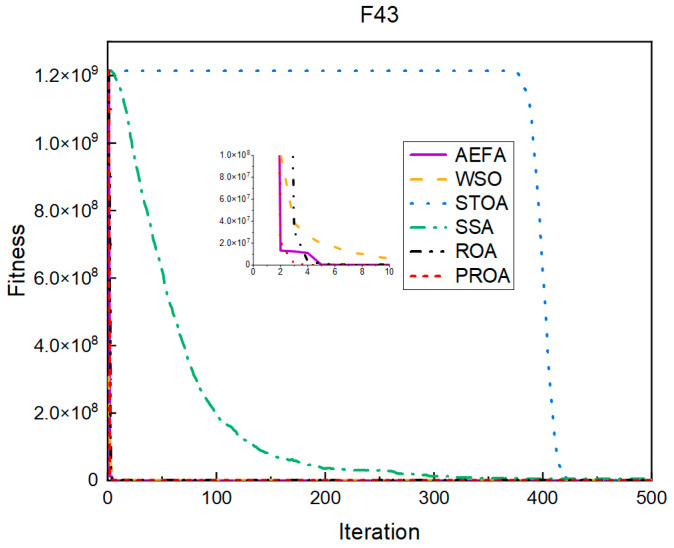
F43’s convergence curve (D = 100).

**Figure 15:**
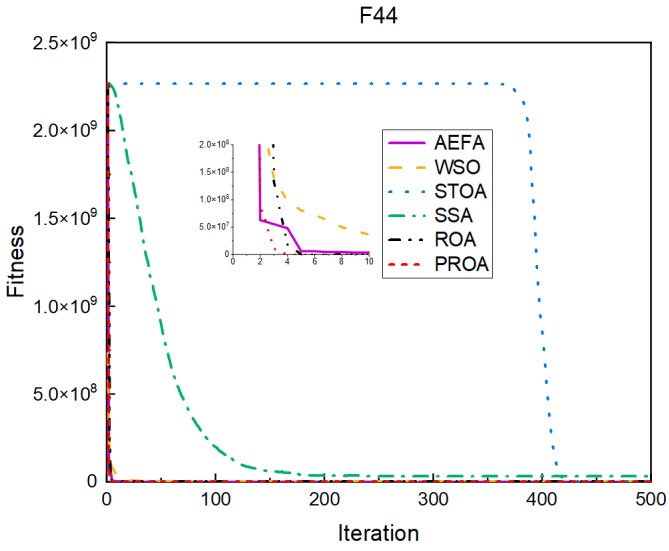
F44’s convergence curve (D = 100).

From the experimental results in Figure 10, Figure 11, Figure 12, Figure 13, Figure 14 and Figure 15, it can be observed that the convergence speed of the PROA still has certain advantages when the dimension is 100. The ROA requires two additional iterations to reach the minimum when optimizing F16 and F17. When optimizing F22 and F41, five additional iterations were required. Only when optimizing F43 and F44 is only one additional iteration required to achieve the same results as the PROA.

#### 4.2.3. Comparison of Results under Dimension 500

The experimental results listed in Table 4 are from when the number of optimized parameters was 500. When the PROA optimizes F16, both the mean and standard deviation of the optimized results are reduced to 0.1% of the ROA. When optimizing F17, the PROA results were more common, and the results were only reduced by 1/2. When the PROA and the ROA were optimized for F22 and F41, the same results were achieved. However, better results were obtained when F43 and F44 were optimized. The mean and standard deviation of the ROA when optimizing F43 were 4% and 3%, respectively. When optimizing F44, the PROA achieved 0.1% and the ROA achieved 0.15%, respectively.

**Figure 16:**
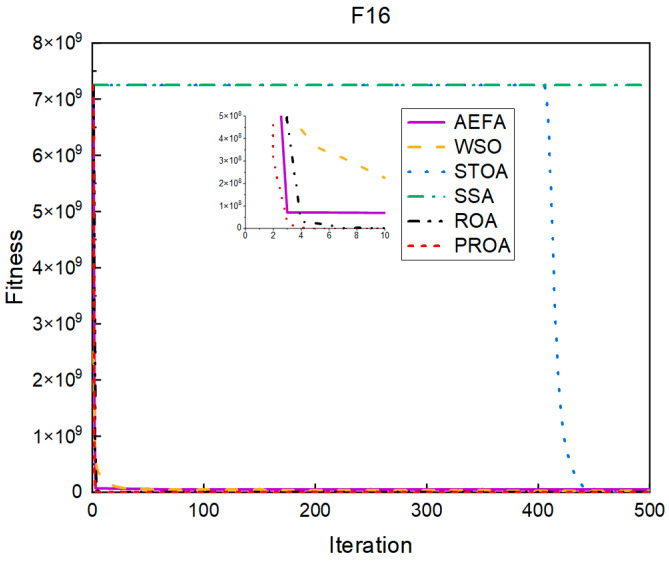
F16’s convergence curve (D = 500).

**Figure 17:**
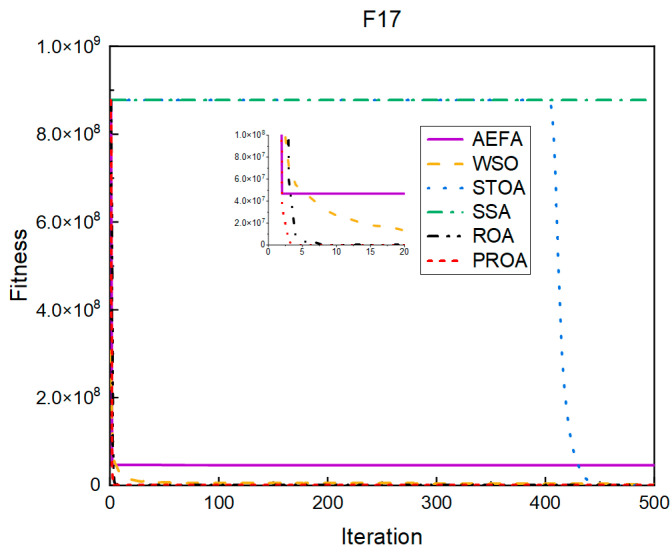
F17’s convergence curve (D = 500).

**Figure 18:**
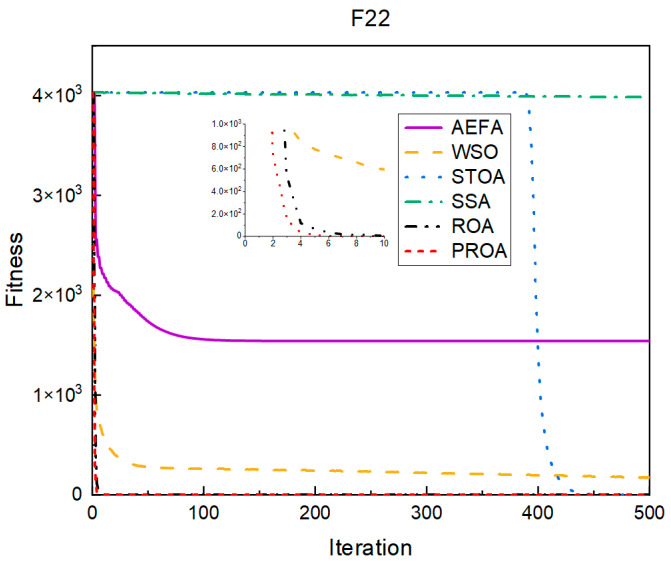
F22’s convergence curve (D = 500).

**Figure 19:**
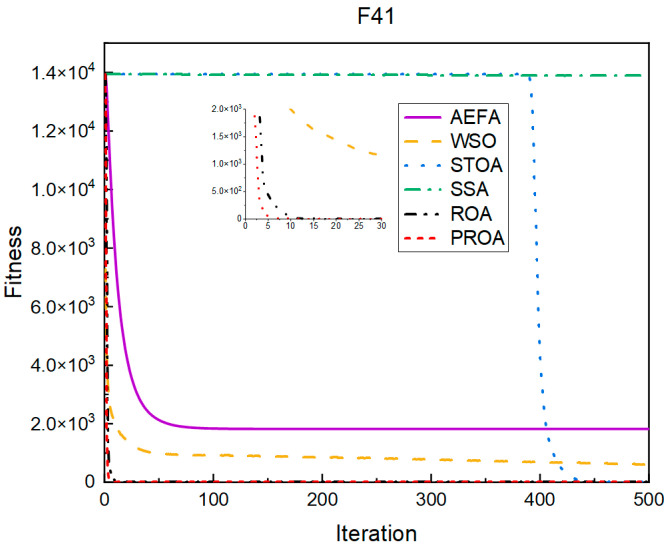
F41’s convergence curve (D = 500).

**Figure 20:**
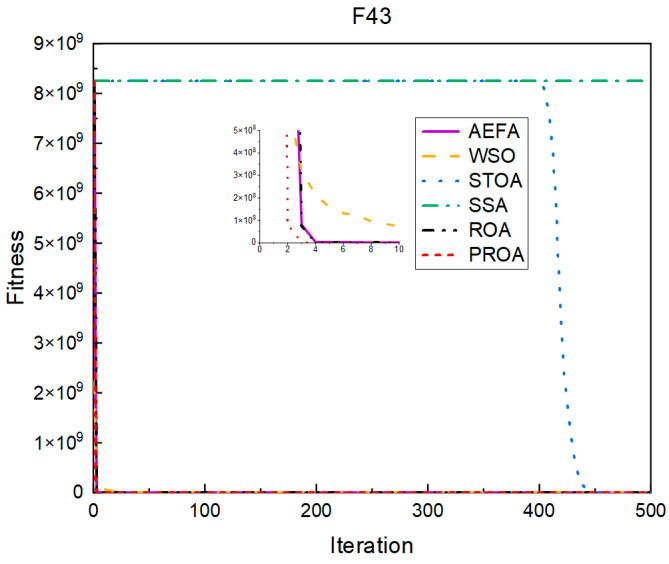
F43’s convergence curve (D = 500).

**Figure 21:**
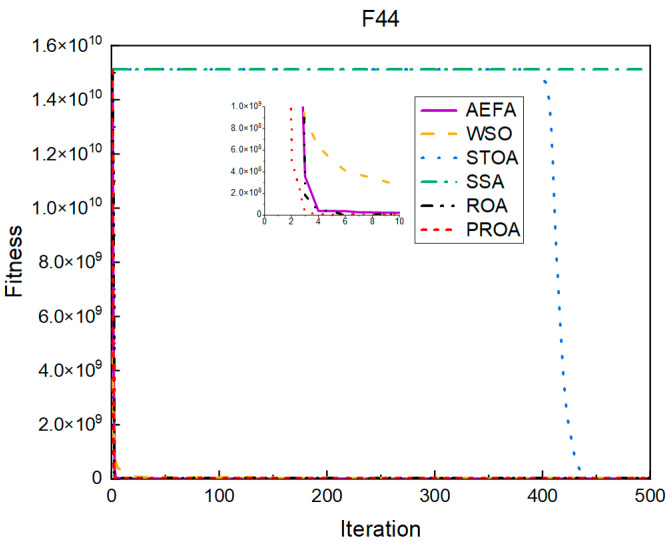
F44’s convergence curve (D = 500).

The convergence when the dimension was 500 is shown in Figure 16, Figure 17, Figure 18, Figure 19, Figure 20 and Figure 21. When the PROA optimized F16, F22, and F44, compared with the ROA, the optimal result was achieved by two iterations ahead of time. While optimizing F17 and F41, it was advanced by five times. When the PROA optimizes F43, the advantage is not obvious, and it only leads to the iterative process in one round.

#### 4.2.4. Comparison of Results under Dimension 1000

The experimental results shown in Table 5 are from when the number of optimized parameters reached 1000. Compared to the ROA, the average optimization result of the PROA was reduced by three orders of magnitude, and the standard deviation was reduced by two orders of magnitude when optimizing F16. However, the PROA’s performance in optimizing F17 was average, the average value only dropped by 1/2, and the standard deviation was similar to that of the ROA. The comparison results of the PROA and the ROA when optimizing F22 and F41 were the same as those of the other dimensions. The PROA obtained better results when F43 and F44 were optimized. In terms of the mean, they were 1% and 0.1% for the ROA results, respectively, and the robustness reached 1% for the ROA.

**Figure 22:**
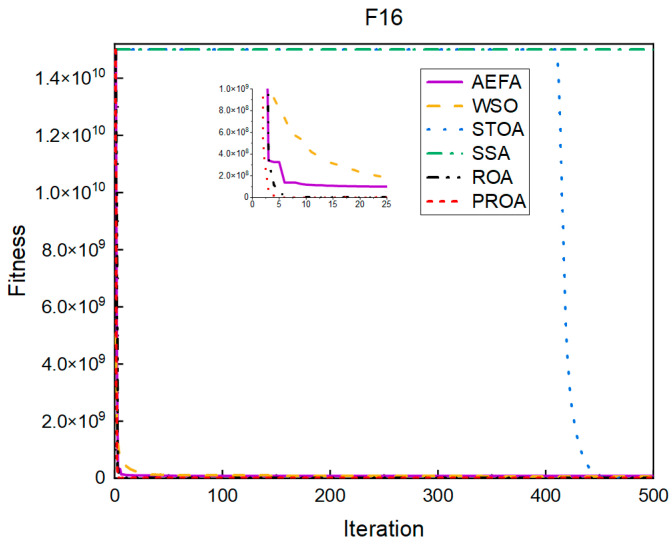
F16’s convergence curve (D = 1000).

**Figure 23:**
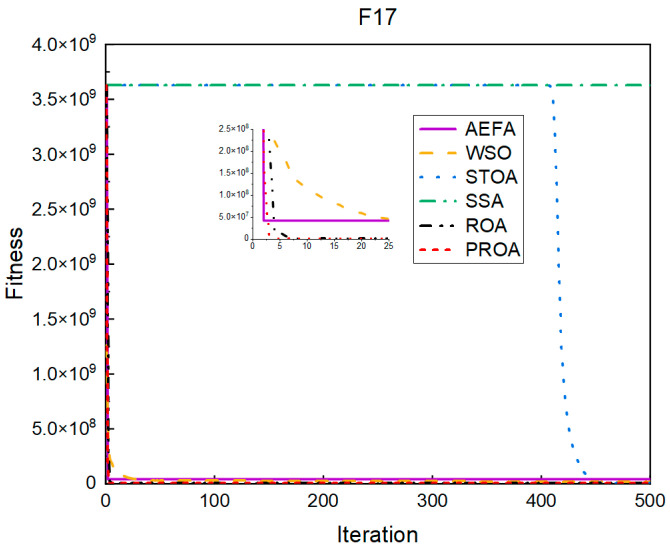
F17’s convergence curve (D = 1000).

**Figure 24:**
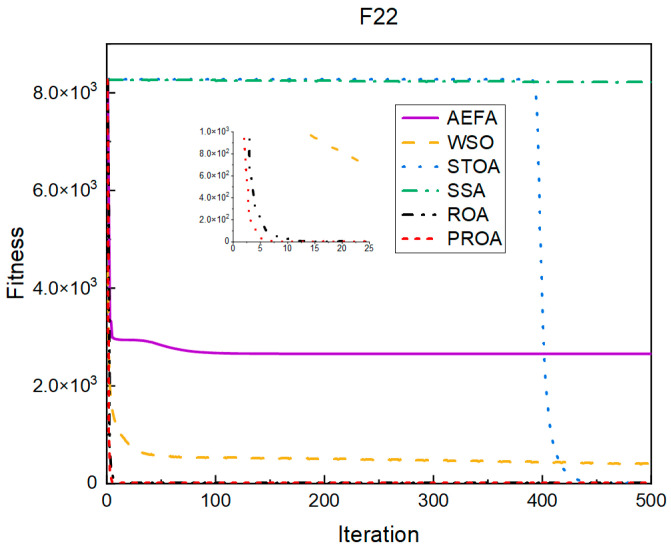
F22’s convergence curve (D = 1000).

**Figure 25:**
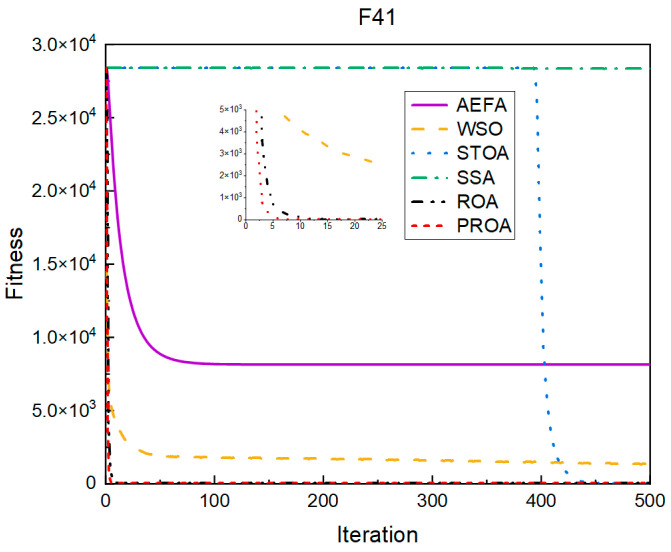
F41’s convergence curve (D = 1000).

**Figure 26:**
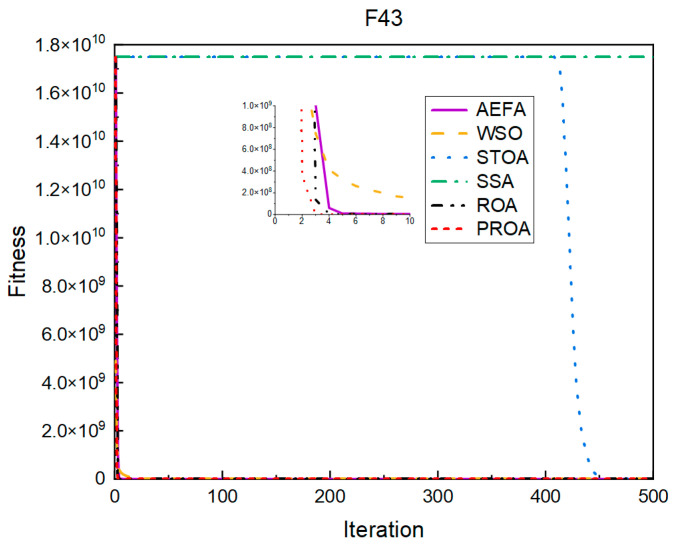
F43’s convergence curve (D = 1000).

**Figure 27:**
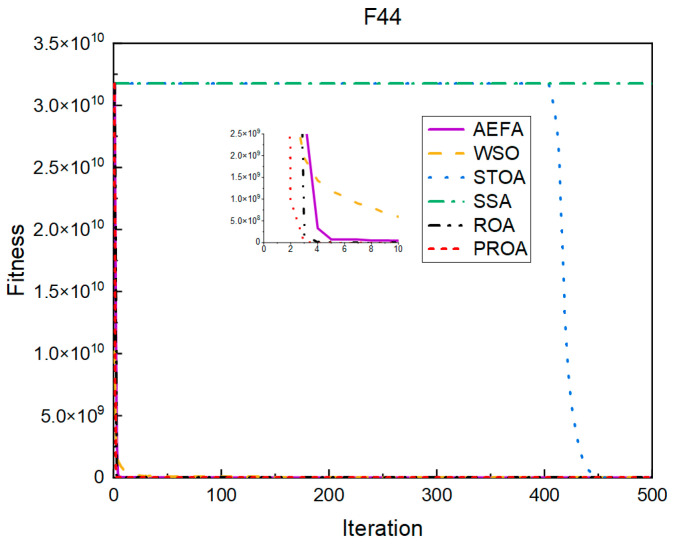
F44’s convergence curve (D = 1000).

The convergence results for 1000 dimensions are shown in Figure 22, Figure 23, Figure 24, Figure 25, Figure 26 and Figure 27. From the experimental results, it can be observed that, compared with the ROA, the PROA obtained the optimal result by two iterations ahead of time when optimizing F16. Three iterations were advanced for optimizing F17 and F41. However, the PROA reached its minimum value with only five iterations when optimizing F22, which was five times that of the ROA. The PROA achieved average results in optimizing F43 and F44, leading to only one iteration.

### 4.3. Results, Statistics, and Performance Analysis

**Figure 28:**
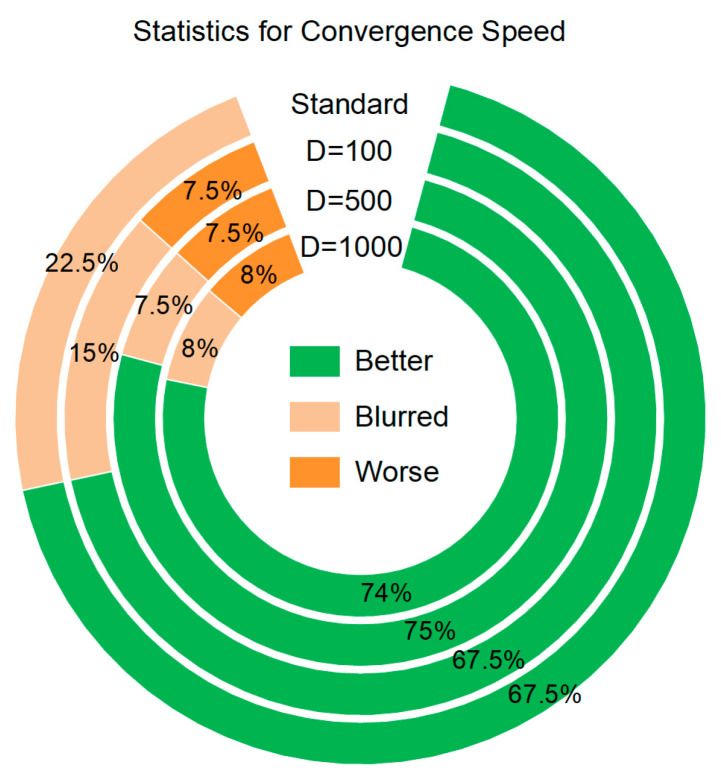
Statistics for convergence speed.

According to the convergence curve (refer to Supplementary Materials for details), we calculated the convergence rate statistics, as shown in Figure 28. Each ring represents the experimental result of one dimension; green indicates that the PROA has a better convergence curve than the ROA; light yellow indicates that the convergence speed of the two algorithms is ambiguous; and orange indicates that the convergence speed of the PROA is worse than that of the ROA. From the statistical results of the experiment, it can be observed that the PROA convergence speed changes on the CEC benchmark function by 67.5–74%. In all dimensions, the rate of slower convergence was 7.5–8%. In addition, there are cases in which the convergence curves of the ROA and the PROA are entangled with each other, but the results of high-dimensional tests are much better than those of standard dimensions.

**Figure 29:**
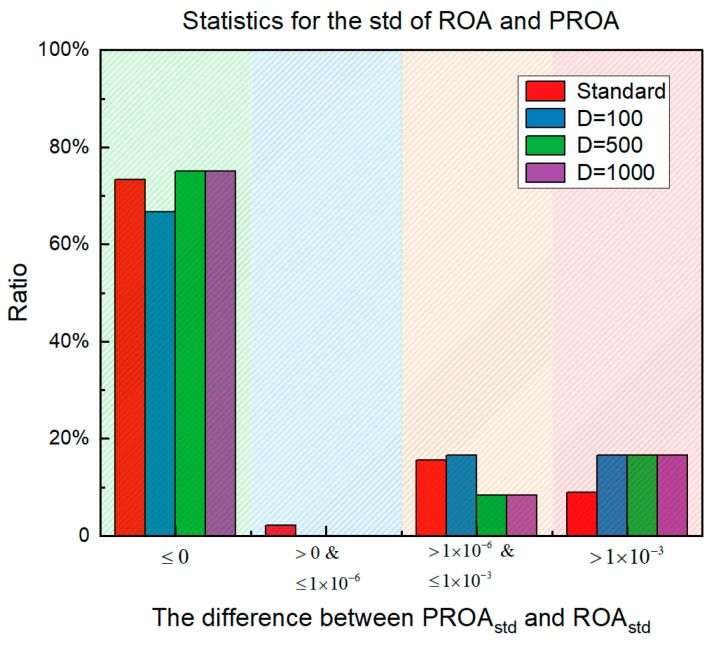
Statistics for the std.

The statistical results based on the standard deviation obtained from the experiments (refer to Supplementary Materials for details) are shown in Figure 29. The horizontal axis represents the difference between the standard deviations of the PROA and the ROA and the vertical axis represents the proportion of the difference in the overall results. It can be observed from the figure that better results than the original ROA were obtained on approximately 75% of the CEC benchmark function. In the test results for the standard dimension, the proportion of performance degradation was less than 9%. In addition, the part whose standard deviation from the ROA was less than 10^–6^ only accounted for 0–2%. However, more than 10^–6^ and less than 10^–3^ accounted for 8–16%.

The experimental results show that the PROA can converge faster than the original ROA in most CEC benchmark functions, whether it is a standard dimension or a high-dimensional parameter. This is because, in the global search stage, the PROA speeds up the search speed and improves the search ability through the Poisson-like randomness strategy, making it more directional than the original ordinary random. Furthermore, the subsequent augmented randomness strategy enables individuals of the population to reach more hosts during the free travel phase. The local search ability near the optimal solution and the overall robustness of the algorithm are enhanced.

## 5. PROA Applied to Deployment Planning

### 5.1. Deployment Planning Model

In large-scale measurements, the target to be measured must have many features and a wide distribution range. One station cannot measure all feature points. Therefore, multiple stations must be determined for planning and measurement. Adjacent stations require at least three public transfer points to complete the coordinate system fitting. With an increase in the number of transfer points, the fitting variance of the coordinate system decreases continuously, but when the number exceeds seven, the reduction speed of the error slows down. Therefore, in the actual measurement process, 5–7 public transfer points are selected for measurement, and the coordinate system is fitted [37]. The following principles should be followed in the deployment of the measuring instruments [38]:

1. A single station can directly measure most features and cover tooling or ground transfer points as much as possible. Simultaneously, priority should be given to selecting transfer points with a large distance and at the edge of the venue.
2. The location of the station should avoid areas with frequent changes in temperature and airflow. Excessive fluctuations directly affected the measurement accuracy of the entire measurement field.
3. The accuracy of the measuring instrument is closely related to the measurement distance. In the establishment of the range, minimizing the distance between the station and the feature to be measured can reduce the measurement error.
4. In the case of tool occlusion, the sum of the fields of view of all the stations should be as large as possible and enclose the entire measurement space.

**Figure 30:**
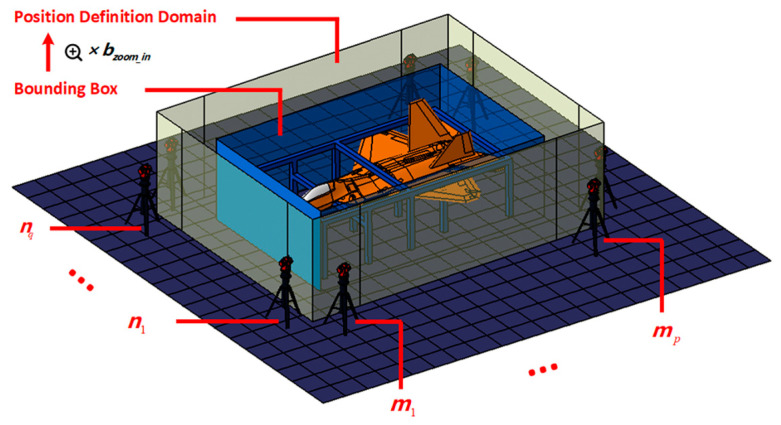
Divide domain.

Based on these principles, it is necessary to first set the planning range of the station. For the target to be measured, the side of the bounding box is the limit planning range, and there is a risk of bumping parts into the arrangement of the measuring equipment. Therefore, the bounding box is first enlarged by $b_{zoom\_in}$, and then the side is divided into *k* areas, where q=⌊k/4⌋, p=⌈k/4⌉. As shown in Figure 30, the translucent blue area is the bounding box of the target to be measured, and the yellow translucent area is the enlarged bounding box based on the original bounding box, which is also the definition domain of the measurement device.

Next, we converted the principles that need to be followed for site deployment into a mathematical model. Station deployment has the following constraints:

1. Between two adjacent stations, it can be observed that the number of public transfer points on the target to be tested cannot be less than $c_{1}$, that is, the constraint $C_{1}\left(x\right)\geq c_{1}$.
2. The number of public transfer points on the tooling that can be observed between two adjacent stations cannot be less than $c_{2}$, that is, constraint $C_{2}\left(x\right)\geq c_{2}$.
3. The number of public transfer points on the ground between two adjacent stations cannot be less than $c_{3}$, that is, the constraint $C_{3}\left(x\right)\geq c_{3}$.
4. The number of reference points that can be observed from all stations should account for above $c_{4}$ of the total number of key points, that is, the constraint $C_{4}\left(x\right)\geq c_{4}$.

Here, $c_{1}$, $c_{2}$ and $c_{3}$ are integers and $c_{4}$ is a decimal in the interval [0,1]. $C_{1}\left(x\right)$, $C_{2}\left(x\right)$, $C_{3}\left(x\right)$, and $C_{4}\left(x\right)$ are constraint functions. All of the constraints filter the visible part of the object under test using a “hidden” point removal operator [39]. The constraint calculation formula is as follows:

(12) $$C\left(x\right)=\{\begin{array}{cc} c-C\left(x\right), & C\left(x\right)<c\\ 0, & C\left(x\right)\geq c \end{array},$$

where c is the constraint value and C(x) is the constraint function.

Finally, the objective function of the deployment model is set. The ultimate goal of this placement model is to minimize the invisible area when it is obscured by tooling. Because the model has multiple constraints, we introduced a large penalty factor σ according to the characteristics of the outlier penalty function. The objective function is then expressed as:

(13) $$F\left(x\right)=1-V_{ratio}+\sigma \times \sum_{i=1}^{4}C_{i}^{2}\left(x\right)$$

(14) $$V_{ratio}=\cup_{j=1}^{k}V_{j}/V_{total}$$

Here, $V_{j}$ is the visible point cloud seen by the j-th station, $V_{ratio}$ is the overall point cloud of the object to be tested, $V_{total}$ is the ratio of the visible area to the total area, and $C_{i}$ is the station constraint.

### 5.2. Simulation Results and 3D Visualization

**Figure 31:**
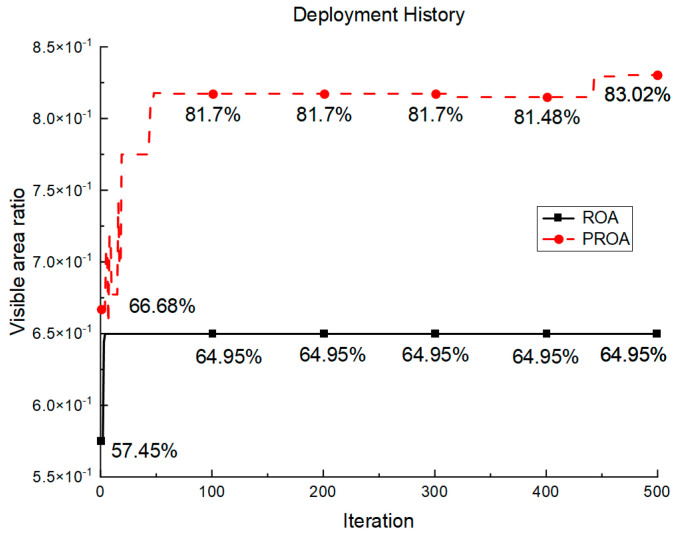
Deployment history.

To verify the feasibility of the station deployment model and the stability of accessibility, the text runs 30 times with the ROA and the PROA as the optimizers under the configuration in Table 6. In addition, in the simulation experiment, the number of target point clouds to be measured, tooling point clouds, and ground point clouds were 51,433, 30,100, and 6847, respectively. Figure 31 shows the average historical results of the maximum visible area of the deployment plan, where the horizontal axis is the number of iterations and the vertical axis is the area ratio of the visible area. The statistical results of all of the experiments are shown in Table 7.

From the experimental results shown in Figure 31, it can be observed that, from the 10th iteration, the ROA convergence speed becomes slower. At the 100th iteration, the maximum visible area obtained by the ROA optimization was 64.95%, whereas the PROA reached 81.7% after rapid convergence. In the subsequent iteration interval of 100–500, the ROA optimization trend tends to be stable. The PROA increased to 83.02%, an increase of 1.32% within this range. When the final iteration completed the entire optimization process, the performance of the PROA was 18.07% higher than that of the ROA.

Conversely, as shown in the statistical results in Table 7, the results of the maximum visible area obtained by the PROA optimization are better than the ROA in three aspects: maximum value, minimum value, and mean value. In terms of robustness, the PROA stability was improved by 1/3. The simulation experiment verifies that the PROA is superior to the ROA, in terms of both convergence speed and robustness.

**Figure 32:**
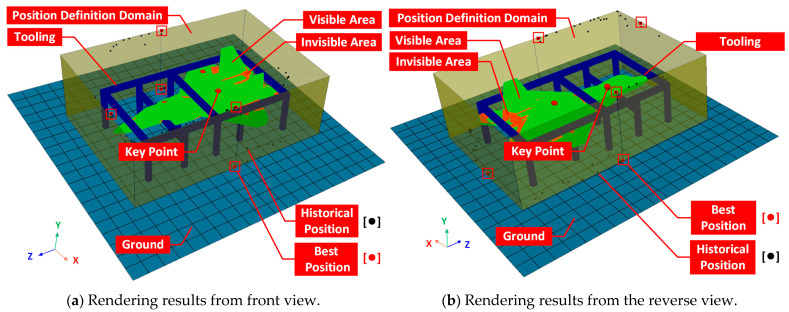
Three-dimensional search result of deployment.

Finally, we used PyVista to render the station’s historical and optimal positions in a 3D space, as shown in Figure 32. The light blue grid is the ground, translucent brown is the station definition domain, dark blue is tooling, red sphere is the key point, green is the visible area, orange is the invisible area, black dots are the historical positions of population exploration during the optimization process, and the red point marked with a red box is the optimal position of the deployment plan after the iteration has been completed.

## 6. Conclusions

In this paper, we propose the PROA. The algorithm introduces a Poisson-like randomness strategy to enhance the global search ability of individual populations. Simultaneously, an enhanced randomness strategy is introduced to improve the local search ability of the population and the robustness of the algorithm. The ROA and PROA were tested with different dimensions (D = standard/100/500/1000) using the CEC benchmark function. The convergence curve results of 67.5–74% of the PROA are better than those of the ROA, and the robustness results of 66.67–75% are better than those of ROA. This study establishes a deployment optimization model for a large-scale measurement field layout planning problem. The PROA was applied to the deployment planning model, and the performance of PROA and the feasibility of the model were verified through simulation experiments. Compared to the ROA, the performance improved by 18.07%, and the maximum viewing area of the PROA can reach 83.02%. It improves the computational efficiency and calculates the overall accessibility compared to traditional station planning methods. Next, we will study the deployment optimization model more deeply from the aspects of cooperation target point measurement accuracy and station transfer accuracy and explore more complex location configuration modes to solve the booth optimization problem [40].

## Figures and Tables

**Table 1:** Part of CEC benchmark functions.

No.	Function	D ^1^	Range	Formulation
F16	Rosenbrock	30	[−30, 30]	$f\left(x\right)=\overset{n-1}{\underset{i=1}{\sum }}\left[100{\left(x_{i+1}-x_{i}^{2}\right)}^{2}+{\left(x_{i}-1\right)}^{2}\right]$
F17	Dixon–Price	30	[−10, 10]	$f\left(x\right)={\left(x_{i}-1\right)}^{2}+\overset{n}{\underset{i=2}{\sum }}i{\left(2x_{i}^{2}-x_{i-1}\right)}^{2}$
F22	Rastrigin	30	[−5.12, 5.12]	$f\left(x\right)=\overset{n}{\underset{i=1}{\sum }}\left[x_{i}^{2}-10\cos\left(2\pi x_{i}\right)+10\right]$
F41	Griewank	30	[−600, 600]	$f\left(x\right)=\frac{1}{4000}\overset{n}{\underset{i=1}{\sum }}x_{2}^{2}-\overset{n}{\underset{i=1}{\prod }}\cos\left(\frac{x_{i}}{\sqrt{i}}\right)+1$
F43	Penalized	30	[−50, 50]	$\begin{array}{c} f\left(x\right)=\frac{\pi }{n}\left\{10\sin^{2}\left(\pi y_{1}\right)+\overset{n-1}{\underset{i=1}{\sum }}{\left(y_{i}-1\right)}^{2}\left[1+10\sin^{2}\left(\pi y_{1}\right)\right]+{\left(y_{n}-1\right)}^{2}\right\}\\ +\overset{n}{\underset{i=1}{\sum }}u\left(x_{i},10,100,4\right)\\ y_{i}=1+\frac{1}{4}\left(x_{i}+1\right)\\ u\left(x_{i},a,k,m\right)=\{\begin{array}{cc} k{\left(x_{i}-a\right)}^{m}, & x_{i}>a\\ 0, & -a\leq x_{i}\leq a\\ k{\left(-x_{i}-a\right)}^{m}, & x<-a \end{array} \end{array}$
F44	Penalized2	30	[−50, 50]	$\begin{array}{c} f\left(x\right)=0.1\{\sin^{2}\left(\pi x_{1}\right)+\overset{n-1}{\underset{i=1}{\sum }}{\left(x_{i}-1\right)}^{2}\left[1+\sin^{2}\left(3\pi x_{i+1}\right)\right]\\ \;+{\left(x_{n}-1\right)}^{2}\left[1+\sin^{2}\left(2\pi x_{n}\right)\right]\}+\overset{n}{\underset{i=1}{\sum }}u\left(x_{i},5,100,4\right) \end{array}$

^1^ Dimension of parameters.

**Table 2:** Results of solving CEC benchmark functions (standard D).

Function	Metric	AEFA	WSO	STOA	SSA	ROA	PROA
F16	Mean	5.45 × 10^4^	1.21 × 10^5^	2.85 × 10^1^	3.23 × 10^6^	1.08 × 10^0^	**2.05 × 10^−2^**
	Std	1.25 × 10^5^	1.08 × 10^5^	4.08 × 10^−1^	1.58 × 10^7^	4.18 × 10^0^	**5.20 × 10^−2^**
F17	Mean	2.87 × 10^3^	8.46 × 10^2^	6.73 × 10^−1^	5.63 × 10^4^	3.99 × 10^−1^	**2.49 × 10^−1^**
	Std	1.19 × 10^4^	8.98 × 10^2^	4.06 × 10^−2^	1.02 × 10^5^	2.85 × 10^−1^	**4.18 × 10^−3^**
F22	Mean	1.15 × 10^0^	2.17 × 10^0^	**0.00 × 10^0^**	7.86 × 10^0^	**0.00 × 10^0^**	**0.00 × 10^0^**
	Std	2.87 × 10^0^	1.20 × 10^0^	**0.00 × 10^0^**	1.21 × 10^1^	**0.00 × 10^0^**	**0.00 × 10^0^**
F41	Mean	1.66 × 10^1^	7.85 × 10^0^	4.88 × 10^−2^	1.64 × 10^1^	**0.00 × 10^0^**	**0.00 × 10^0^**
	Std	5.84 × 10^0^	4.13 × 10^0^	6.15 × 10^−2^	4.35 × 10^1^	**0.00 × 10^0^**	**0.00 × 10^0^**
F43	Mean	2.67 × 10^1^	3.67 × 10^0^	1.52 × 10^−1^	1.32 × 10^2^	1.69 × 10^−4^	**9.50 × 10^−6^**
	Std	9.25 × 10^0^	4.90 × 10^0^	5.98 × 10^−2^	1.22 × 10^2^	3.80 × 10^−4^	**1.81 × 10^−5^**
F44	Mean	6.25 × 10^2^	1.07 × 10^3^	2.04 × 10^0^	3.04 × 10^3^	6.76 × 10^−3^	**4.68 × 10^−5^**
	Std	2.03 × 10^2^	2.72 × 10^3^	2.44 × 10^−1^	7.28 × 10^2^	1.35 × 10^−2^	**1.55 × 10^−4^**

**Table 3:** Results of solving CEC benchmark functions (D = 100).

Function	Metric	AEFA	WSO	STOA	SSA	ROA	PROA
F16	Mean	6.68 × 10^6^	1.58 × 10^6^	1.10 × 10^2^	4.17 × 10^8^	1.38 × 10^1^	**8.67 × 10^−2^**
	Std	2.57 × 10^6^	7.11 × 10^5^	1.46 × 10^1^	3.75 × 10^8^	3.19 × 10^1^	**1.80 × 10^−1^**
F17	Mean	1.41 × 10^6^	3.70 × 10^4^	1.42 × 10^0^	3.71 × 10^6^	6.14 × 10^−1^	**2.54 × 10^−1^**
	Std	4.84 × 10^5^	2.14 × 10^4^	5.09 × 10^−1^	2.06 × 10^6^	3.58 × 10^−1^	**6.42 × 10^−3^**
F22	Mean	1.20 × 10^2^	1.88 × 10^1^	4.34 × 10^−5^	9.55 × 10^1^	**0.00 × 10^0^**	**0.00 × 10^0^**
	Std	3.22 × 10^1^	4.32 × 10^0^	7.00 × 10^−5^	3.88 × 10^1^	**0.00 × 10^0^**	**0.00 × 10^0^**
F41	Mean	1.26 × 10^2^	6.46 × 10^1^	4.80 × 10^−2^	3.84 × 10^2^	**0.00 × 10^0^**	**0.00 × 10^0^**
	Std	1.69 × 10^1^	1.34 × 10^1^	6.57 × 10^−2^	1.41 × 10^2^	**0.00 × 10^0^**	**0.00 × 10^0^**
F43	Mean	2.42 × 10^2^	9.56 × 10^2^	3.96 × 10^−1^	5.12 × 10^6^	2.70 × 10^−4^	**5.84 × 10^−6^**
	Std	6.28 × 10^2^	5.54 × 10^3^	5.02 × 10^−1^	3.62 × 10^7^	1.43 × 10^−3^	**1.24 × 10^−5^**
F44	Mean	7.87 × 10^4^	1.64 × 10^5^	1.10 × 10^1^	3.28 × 10^7^	1.17 × 10^−1^	**7.19 × 10^−5^**
	Std	1.19 × 10^5^	2.59 × 10^5^	7.92 × 10^−1^	1.12 × 10^8^	5.78 × 10^−1^	**8.77 × 10^−5^**

**Table 4:** Results of solving CEC benchmark functions (D = 500).

Function	Metric	AEFA	WSO	STOA	SSA	ROA	PROA
F16	Mean	4.94 × 10^7^	2.03 × 10^7^	2.04 × 10^4^	7.24 × 10^9^	1.32 × 10^2^	**2.61 × 10^−1^**
	Std	7.66 × 10^6^	4.27 × 10^6^	1.87 × 10^4^	2.29 × 10^8^	1.96 × 10^2^	**3.29 × 10^−1^**
F17	Mean	4.59 × 10^7^	2.42 × 10^6^	2.74 × 10^3^	8.77 × 10^8^	8.16 × 10^−1^	**3.37 × 10^−1^**
	Std	4.95 × 10^6^	6.15 × 10^5^	2.05 × 10^3^	3.49 × 10^7^	3.20 × 10^−1^	**1.81 × 10^−1^**
F22	Mean	1.54 × 10^3^	1.71 × 10^2^	2.72 × 10^−2^	3.98 × 10^3^	**0.00 × 10^0^**	**0.00 × 10^0^**
	Std	1.08 × 10^2^	1.71 × 10^1^	2.53 × 10^−2^	1.02 × 10^2^	**0.00 × 10^0^**	**0.00 × 10^0^**
F41	Mean	1.82 × 10^3^	6.01 × 10^2^	2.94 × 10^−1^	1.39 × 10^4^	**0.00 × 10^0^**	**0.00 × 10^0^**
	Std	6.10 × 10^1^	5.11 × 10^1^	2.04 × 10^−1^	2.70 × 10^2^	**0.00 × 10^0^**	**0.00 × 10^0^**
F43	Mean	2.75 × 10^5^	2.13 × 10^5^	5.12 × 10^0^	8.25 × 10^9^	1.18 × 10^−4^	**5.00 × 10^−6^**
	Std	2.63 × 10^5^	2.15 × 10^5^	4.48 × 10^0^	6.39 × 10^8^	3.04 × 10^−4^	**1.06 × 10^−5^**
F44	Mean	8.40 × 10^6^	4.99 × 10^6^	3.83 × 10^2^	1.51 × 10^10^	2.98 × 10^−1^	**5.35 × 10^−4^**
	Std	3.12 × 10^6^	3.17 × 10^6^	2.87 × 10^2^	9.20 × 10^8^	7.00 × 10^−1^	**1.05 × 10^−3^**

**Table 5:** Results of solving CEC benchmark functions (D = 1000).

Function	Metric	AEFA	WSO	STOA	SSA	ROA	PROA
F16	Mean	8.39 × 10^7^	5.33 × 10^7^	3.09 × 10^5^	1.50 × 10^10^	1.82 × 10^2^	**6.81 × 10^−1^**
	Std	8.79 × 10^6^	1.06 × 10^7^	4.38 × 10^5^	2.93 × 10^8^	3.35 × 10^2^	**1.09 × 10^0^**
F17	Mean	4.24 × 10^7^	1.30 × 10^7^	8.78 × 10^4^	3.63 × 10^9^	9.07 × 10^−1^	**4.89 × 10^−1^**
	Std	3.96 × 10^6^	2.49 × 10^6^	8.20 × 10^4^	1.01 × 10^8^	**2.27 × 10^−1^**	2.91 × 10^−1^
F22	Mean	2.66 × 10^3^	3.92 × 10^2^	1.47 × 10^−1^	8.21 × 10^3^	**0.00 × 10^0^**	**0.00 × 10^0^**
	Std	1.20 × 10^2^	3.76 × 10^1^	1.08 × 10^−1^	1.48 × 10^2^	**0.00 × 10^0^**	**0.00 × 10^0^**
F41	Mean	8.15 × 10^3^	1.32 × 10^3^	8.81 × 10^−1^	2.83 × 10^4^	**0.00 × 10^0^**	**0.00 × 10^0^**
	Std	1.42 × 10^2^	1.48 × 10^2^	4.75 × 10^−1^	4.13 × 10^2^	**0.00 × 10^0^**	**0.00 × 10^0^**
F43	Mean	1.14 × 10^6^	1.13 × 10^6^	3.13 × 10^2^	1.75 × 10^10^	1.01 × 10^−4^	**2.16 × 10^−6^**
	Std	6.52 × 10^5^	1.30 × 10^6^	2.10 × 10^3^	6.93 × 10^8^	1.83 × 10^−4^	**3.84 × 10^−6^**
F44	Mean	2.42 × 10^7^	1.65 × 10^7^	2.72 × 10^3^	3.18 × 10^10^	2.18 × 10^−1^	**9.81 × 10^−4^**
	Std	4.93 × 10^6^	5.46 × 10^6^	2.52 × 10^3^	1.32 × 10^9^	4.93 × 10^−1^	**1.83 × 10^−3^**

**Table 6:** Simulation configuration.

No.	Parameter	Symbol	Value
1	Key point (xyz)	/	220, 24, −640 −230, 24, −640 19, 62, −590 −19, 60, −290
2	Number of laser trackers	k	6
3	Bounding box magnification factor	$b_{zoom\_in}$	0.2
4	Number of populations	NP	20
5	Maximum number of iterations	max_iter	500
6	The number of public transfer points on the target to be tested can be seen between two adjacent stations	$c_{1}$	2
7	The number of public transfer points on the tooling can be seen between two adjacent stations	$c_{2}$	1
8	The number of public transfer points seen on the ground between two adjacent stations	$c_{3}$	2
9	The proportion of the number of key points that can be seen in all stations	$c_{4}$	0.75
10	Penalty factor	σ	106

**Table 7:** Deployment result (30 times).

Algorithm	Min	Max	Mean	Std
ROA	64.95%	80.18%	75.82%	0.0350
PROA	**73.51%**	**83.02%**	**79.63%**	**0.0225**

## Data Availability

Owing to the large dataset, we only uploaded the entire code to GitHub at https://github.com/YDM-Cloud/PROA. The dataset in this study is available on request from the corresponding author.

## Supplementary Materials

The following supporting information can be downloaded at: https://github.com/YDM-Cloud/PROA/tree/main/documents.

## References

[1] Muelaner J.E., Wang Z., Martin O., Jamshidi J., Maropoulos P.G. (2010). Estimation of uncertainty in three-dimensional coordinate measurement by comparison with calibrated points. Meas. Sci. Technol..

[2] Suthunyatanakit K., Bohez E.L., Annanon K. (2009). A new global accessibility algorithm for a polyhedral model with convex polygonal facets. Comput. Des..

[3] Nuñez A., Lacasa L., Valero E., Gómez J.P., Luque B. (2012). Detecting series periodicity with horizontal visibility graphs. Int. J. Bifurc. Chaos.

[4] Lin X. (2016). Based on the Full 3D Model, the Measurement Method and Experimental Research of Large Aircraft Parts Assembly Docking. Doctoral Dissertation.

[5] Dong Y., Cao L., Zuo K. (2022). Genetic algorithm based on a new similarity for probabilistic transformation of belief functions. Entropy.

[6] Wan C., He B., Fan Y., Tan W., Qin T., Yang J. (2022). Improved black widow spider optimization algorithm integrating multiple strategies. Entropy.

[7] Wu F., Zhang J., Li S., Lv D., Li M. (2022). An enhanced differential evolution algorithm with bernstein operator and refracted oppositional-mutual learning strategy. Entropy.

[8] Pang S., Liu J., Zhang Z., Fan X., Zhang Y., Zhang D., Hwang G.H. (2022). A photovoltaic power predicting model using the differential evolution algorithm and multi-task learning. Front. Mater..

[9] Opoku E., Ahmed S., Song Y., Nathoo F. (2021). Ant colony system optimization for spatiotemporal modelling of combined EEG and MEG data. Entropy.

[10] Wu L., Qu J., Shi H., Li P. (2022). Node deployment optimization for wireless sensor networks based on virtual force-directed particle swarm optimization algorithm and evidence theory. Entropy.

[11] Dai T., Miao L., Shao H., Shi Y. (2019). Solving gravity anomaly matching problem under large initial errors in gravity aided navigation by using an affine transformation based artificial bee colony algorithm. Front. Neurorobotics.

[12] Dong Z., Zheng J., Huang S., Pan H., Liu Q. (2019). Time-shift multi-scale weighted permutation entropy and GWO-SVM based fault diagnosis approach for rolling bearing. Entropy.

[13] Zhou J., Guo X., Wang Z., Du W., Han X., He G., Xue H., Kou Y. (2019). Research on fault extraction method of variational mode decomposition based on immunized fruit fly optimization algorithm. Entropy.

[14] Lu S., Wang S.-H., Zhang Y.-D. (2021). Detection of abnormal brain in MRI via improved AlexNet and ELM optimized by chaotic bat algorithm. Neural Comput. Appl..

[15] Tong Y., Yu B. (2022). Research on hyper-parameter optimization of activity recognition algorithm based on improved cuckoo search. Entropy.

[16] Deb S., Gao X.-Z., Tammi K., Kalita K., Mahanta P. (2020). Recent studies on chicken swarm optimization algorithm: A review (2014–2018). Artif. Intell. Rev..

[17] Kuo C.L., Kuruoglu E.E., Chan W.K.V. (2022). Neural network structure optimization by simulated annealing. Entropy.

[18] Shang R., Zhang W., Li F., Jiao L., Stolkin R. (2019). Multi-objective artificial immune algorithm for fuzzy clustering based on multiple kernels. Swarm Evol. Comput..

[19] Liao Y., Liu Y., Chen C., Zhang L. (2021). Green building energy cost optimization with deep belief network and firefly algorithm. Front. Energy Res..

[20] Goh R., Lee L., Seow H.-V., Gopal K. (2020). Hybrid harmony search—Artificial intelligence models in credit scoring. Entropy.

[21] Jia H., Peng X., Lang C. (2021). Remora optimization algorithm. Expert Syst. Appl..

[22] Mirjalili S., Lewis A. (2016). The whale optimization algorithm. Adv. Eng. Softw..

[23] Shadravan S., Naji H., Bardsiri V. (2019). The sailfish optimizer: A novel nature-inspired metaheuristic algorithm for solving constrained engineering optimization problems. Eng. Appl. Artif. Intell..

[24] Almalawi A., Khan A.I., Alqurashi F., Abushark Y.B., Alam M., Qaiyum S. (2022). Modeling of remora optimization with deep learning enabled heavy metal sorption efficiency prediction onto biochar. Chemosphere.

[25] Raamesh L., Radhika S., Jothi S. (2022). A cost-effective test case selection and prioritization using hybrid battle royale-based remora optimization. Neural Comput. Appl..

[26] Chou J.-S., Nguyen N.-M. (2020). FBI inspired meta-optimization. Appl. Soft Comput..

[27] Anita, Yadav A. (2019). AEFA: Artificial electric field algorithm for global optimization. Swarm Evol. Comput..

[28] Braik M., Hammouri A., Atwan J., Al-Betar M.A., Awadallah M.A. (2022). White shark optimizer: A novel bio-inspired meta-heuristic algorithm for global optimization problems. Knowl.-Based Syst..

[29] Dhiman G., Kaur A. (2019). STOA: A bio-inspired based optimization algorithm for industrial engineering problems. Eng. Appl. Artif. Intell..

[30] Jain M., Singh V., Rani A. (2019). A novel nature-inspired algorithm for optimization: Squirrel search algorithm. Swarm Evol. Comput..

[31] Tan C., Chang S., Liu L. (2017). Hierarchical genetic-particle swarm optimization for bistable permanent magnet actuators. Appl. Soft Comput..

[32] Qiao W., Yang Z. (2019). An improved dolphin swarm algorithm based on kernel fuzzy C-means in the application of solving the optimal problems of large-scale function. IEEE Access.

[33] Wang H., Jin Y., Doherty J. (2017). Committee-based active learning for surrogate-assisted particle swarm optimization of expensive problems. IEEE Trans. Cybern..

[34] Kan G., Zhang M., Liang K., Wang H., Jiang Y., Li J., Ding L., He X., Hong Y., Zuo D. (2018). Improving water quantity simulation & forecasting to solve the energy-water-food nexus issue by using heterogeneous computing accelerated global optimization method. Appl. Energy.

[35] Chen K., Zhou F., Liu A. (2018). Chaotic dynamic weight particle swarm optimization for numerical function optimization. Knowl.-Based Syst..

[36] Samma H., Sama A.S.B. (2022). Rules embedded harris hawks optimizer for large-scale optimization problems. Neural Comput. Appl..

[37] Prasad S., Kumar D.V. (2018). Trade-offs in PMU and IED deployment for active distribution state estimation using multi-objective evolutionary algorithm. IEEE Trans. Instrum. Meas..

[38] Agrawal B.N., Platzer M.F. (2018). Standard Handbook for Aerospace Engineers.

[39] Katz S., Tal A., Basri R. (2007). Direct visibility of point sets. ACM SIGGRAPH 2007 Papers.

[40] Li Y., Zhang X., Zhao J., Yang X., Xi M. (2022). Position deployment optimization of maneuvering conventional missile based on improved whale optimization algorithm. Int. J. Aerosp. Eng..

